# Erector Spinae Muscle to Epicardial Visceral Fat Ratio on Chest CT Predicts the Severity of Coronavirus Disease 2019

**DOI:** 10.1002/jcsm.13721

**Published:** 2025-01-27

**Authors:** Takashi Shimada, Tomoki Maetani, Shotaro Chubachi, Naoya Tanabe, Takanori Asakura, Ho Namkoong, Hiromu Tanaka, Shuhei Azekawa, Shiro Otake, Kensuke Nakagawara, Takahiro Fukushima, Mayuko Watase, Yusuke Shiraishi, Hideki Terai, Mamoru Sasaki, Soichiro Ueda, Yukari Kato, Norihiro Harada, Shoji Suzuki, Shuichi Yoshida, Hiroki Tateno, Kaoruko Shimizu, Susumu Sato, Yoshitake Yamada, Masahiro Jinzaki, Toyohiro Hirai, Yukinori Okada, Ryuji Koike, Makoto Ishii, Akinori Kimura, Seiya Imoto, Satoru Miyano, Seishi Ogawa, Takanori Kanai, Koichi Fukunaga

**Affiliations:** ^1^ Division of Pulmonary Medicine, Department of Medicine Keio University School of Medicine Tokyo Japan; ^2^ Department of Respiratory Medicine, Graduate School of Medicine Kyoto University Kyoto Japan; ^3^ Department of Clinical Medicine (Laboratory of Bioregulatory Medicine) Kitasato University School of Pharmacy Tokyo Japan; ^4^ Department of Respiratory Medicine, Kitasato University Kitasato Institute Hospital Tokyo Japan; ^5^ Department of Infectious Diseases Keio University School of Medicine Tokyo Japan; ^6^ Internal Medicine JCHO (Japan Community Health Care Organization) Saitama Medical Center Saitama Japan; ^7^ Department of Respiratory Medicine Juntendo University Faculty of Medicine and Graduate School of Medicine Tokyo Japan; ^8^ Department of Pulmonary Medicine Saitama City Hospital Saitama Japan; ^9^ Department of Respiratory Medicine, Graduate School of Medicine Hokkaido University Sapporo Japan; ^10^ Department of Respiratory Care and Sleep Control Medicine, Graduate School of Medicine Kyoto University Kyoto Japan; ^11^ Department of Radiology Keio University School of Medicine Tokyo Japan; ^12^ Department of Statistical Genetics Osaka University Graduate School of Medicine Suita Japan; ^13^ Department of Genome Informatics, Graduate School of Medicine The University of Tokyo Tokyo Japan; ^14^ Laboratory for Systems Genetics RIKEN Center for Integrative Medical Sciences Yokohama Kanagawa Japan; ^15^ Health Science Research and Development Center Tokyo Medical and Dental University Tokyo Japan; ^16^ Department of Respiratory Medicine Nagoya University Graduate School of Medicine Nagoya Japan; ^17^ Institute of Research Tokyo Medical and Dental University Tokyo Japan; ^18^ Division of Health Medical Intelligence, Human Genome Center, Institute of Medical Science University of Tokyo Tokyo Japan; ^19^ M&D Data Science Center Tokyo Medical and Dental University Tokyo Japan; ^20^ Department of Pathology and Tumor Biology Kyoto University Kyoto Japan; ^21^ Institute for the Advanced Study of Human Biology (WPI‐ASHBi) Kyoto University Kyoto Japan; ^22^ Division of Gastroenterology and Hepatology, Department of Internal Medicine Keio University School of Medicine Tokyo Japan

**Keywords:** computed tomography, COVID‐19, epicardial adipose tissue, erector spinae muscles, obesity, sarcopenia

## Abstract

**Background:**

Chest computed tomography (CT) is a valuable tool for diagnosing and predicting the severity of coronavirus disease 2019 (COVID‐19) and assessing extrapulmonary organs. Reduced muscle mass and visceral fat accumulation are important features of a body composition phenotype in which obesity and muscle loss coexist, but their relationship with COVID‐19 outcomes remains unclear. In this study, we aimed to investigate the association between the erector spinae muscle (ESM) to epicardial adipose tissue (EAT) ratio (ESM/EAT) on chest CT and disease severity in patients with COVID‐19.

**Methods:**

We analysed data from 1074 COVID‐19 patients enrolled in the Japan COVID‐19 Task Force database. The primary outcome was the rate of critical outcomes (requiring high‐flow oxygen therapy, invasive ventilator support or death). The incidence of critical outcomes was compared between patients with high and low ESM/EAT ratios.

**Results:**

The low ESM/EAT group (*n* = 353) had a higher incidence of critical outcomes (13.3% vs. 5.13%, *p* < 0.001) and mortality (2.55% vs. 0.69%, *p* = 0.019) than the high ESM/EAT group (*n* = 721). In multivariable analysis, the low ESM/EAT ratio was associated with critical outcomes (adjusted odds ratio [aOR] 2.11, 95% confidence interval [CI] 1.22–3.66) independently of the known COVID‐19 severity factors including age, sex, body mass index (BMI), smoking history, lifestyle‐related comorbidities and pneumonia volume.

**Conclusion:**

The low ESM/EAT ratio in COVID‐19 patients can be obtained on chest CT and used to predict critical outcomes after disease onset, demonstrating the importance of detailed body composition assessments in COVID‐19 practice.

## 1 Introduction

Since December 2019, coronavirus disease 2019 (COVID‐19), caused by the severe acute respiratory syndrome coronavirus 2 (SARS‐CoV‐2), has infected hundreds of millions of people worldwide, with outcomes ranging from asymptomatic to severe or fatal. Identifying patients at risk for severe COVID‐19 remains crucial, as does establishing biomarkers for future pandemics.

A decline in skeletal muscle mass is linked to reduced physical function and increased mortality in various disease [1]. Body composition metrics such as height, weight and body mass index (BMI) do not always provide an accurate assessment of muscle mass, making computed tomography (CT) an important supplementary tool. Chest CT, widely used for diagnosing and assessing the severity of COVID‐19, also enables evaluation of extrapulmonary organs. We have previously shown that indices of extrapulmonary organs correlate with COVID‐19 severity [2, 3, 4]. While some studies have demonstrated an association between low erector spinae muscle (ESM) mass and poor prognosis in COVID‐19 [3], others have found no such relationship [5].

Muscle loss and fat accumulation frequently coexist, but muscle loss is often overlooked in patients with obesity [6]. The muscle‐to‐fat ratio, assessed via CT imaging, has become a key measure for evaluating this condition, with previous studies linking it to prognosis in patients with solid tumours [7] and postoperative complications [8]. However, no studies have yet explored the association between the muscle‐to‐fat ratio and outcomes in COVID‐19 patients. Recently, several studies have successfully utilized chest CT to quantify the muscle‐to‐fat ratio [9, 10]. We hypothesized that this ratio, as measured on chest CT, is associated with poor outcomes in COVID‐19. In this study, we aimed to investigate the relationship between the muscle‐to‐visceral fat ratio (ESM/epicardial adipose tissue [EAT] ratio) and critical outcomes in patients with COVID‐19.

## 2 Methods

### 2.1 Study Design

**FIGURE 1:**
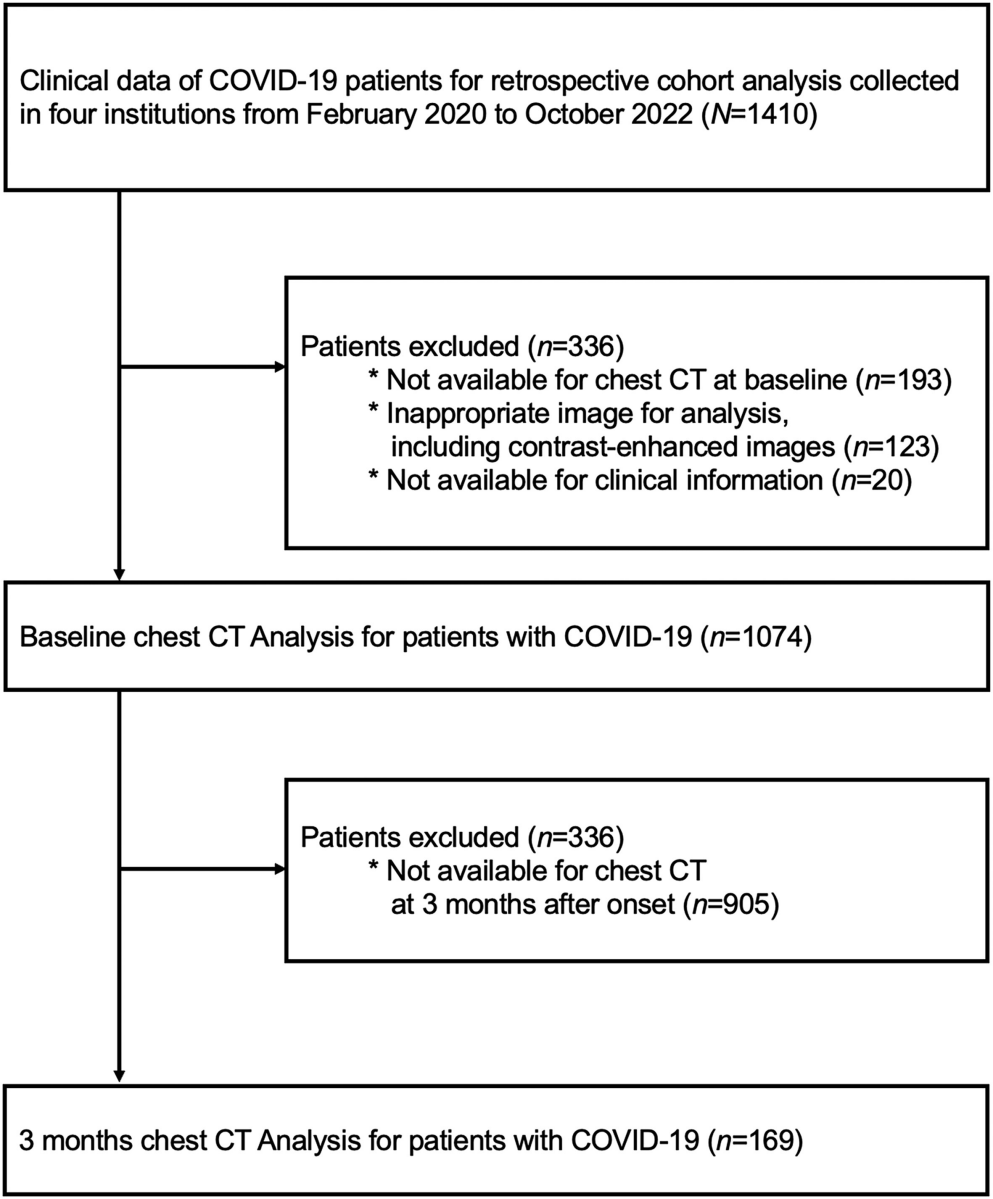
Process of patient selection in this study. Out of the 1410 COVID‐19 hospitalized patients from the four institutions during the study period, 336 were excluded. Therefore, 1074 patients were included in the analysis, of which 169 had 3 months chest CT analysis.

As previously described [2, 3, 11], this retrospective cohort study analysed data from the Japan COVID‐19 Task Force database collected from February 2020 to October 2022. Clinical information was collected from 1410 patients with COVID‐19 from four Japanese institutions (Keio University Hospital, Juntendo University Hospital, Saitama Medical Center and Saitama City Hospital). Among these patients, 336 patients were excluded because of the unavailability of baseline chest CT data (*n* = 193), images inappropriate for analysis, including contrast‐enhanced images (*n* = 123) and unavailability of clinical information (*n* = 20). Thus, 1074 patients were included in the analysis (Figure 1). The study was approved by the Ethics Committee of Keio University School of Medicine (ID: 20200061) and was conducted in accordance with the principles of the Declaration of Helsinki. Written or oral informed consent was obtained from all participants.

### 2.2 Clinical Data

The following clinical data were collected from patients with COVID‐19: age, sex, height, weight, clinical signs and symptoms, laboratory findings on admission, comorbidities and treatment details. All laboratory and radiological tests were performed within 48 h of the initial visit or admission. The primary outcome of this study was the proportion of patients with critical outcomes, which were defined as the need for oxygen supplementation via high‐flow oxygen therapy, mechanical ventilation, extracorporeal membrane oxygenation or death [12, 13]. For the stratification analysis across each epidemic phase, we defined the epidemic waves as follows: the first wave, from 29 January 2020 to 13 June 2020; the second, from 14 June 2020 to 9 October 2020; the third, from 10 October 2020 to 28 February 2021; the fourth (Alpha variant‐dominated wave), from 1 March 2021 to 20 June 2021; the fifth (Delta variant‐dominated wave), from 21 June 2021 to 16 December 2021; the sixth (Omicron variant‐dominated wave), from 17 December 2021 to 24 June 2022; and the seventh (Omicron variant‐dominated wave), from 25 June 2022 to 26 September 2022, in accordance with previous literature [11].

### 2.3 CT Image Acquisition

All CT images were acquired after complete inspiration. Images of the entire lung with a slice thickness of 1–5 mm were reconstructed using standard kernels. CT images were acquired using the SOMATOM series (Siemens Healthineers), Aquilion series (Canon Medical Systems), Revolution series (GE Healthcare), Discovery series (GE Healthcare) and BrightSpeed (GE Healthcare).

### 2.4 CT Analysis of Pulmonary and Extrapulmonary Lesions

**FIGURE 2:**
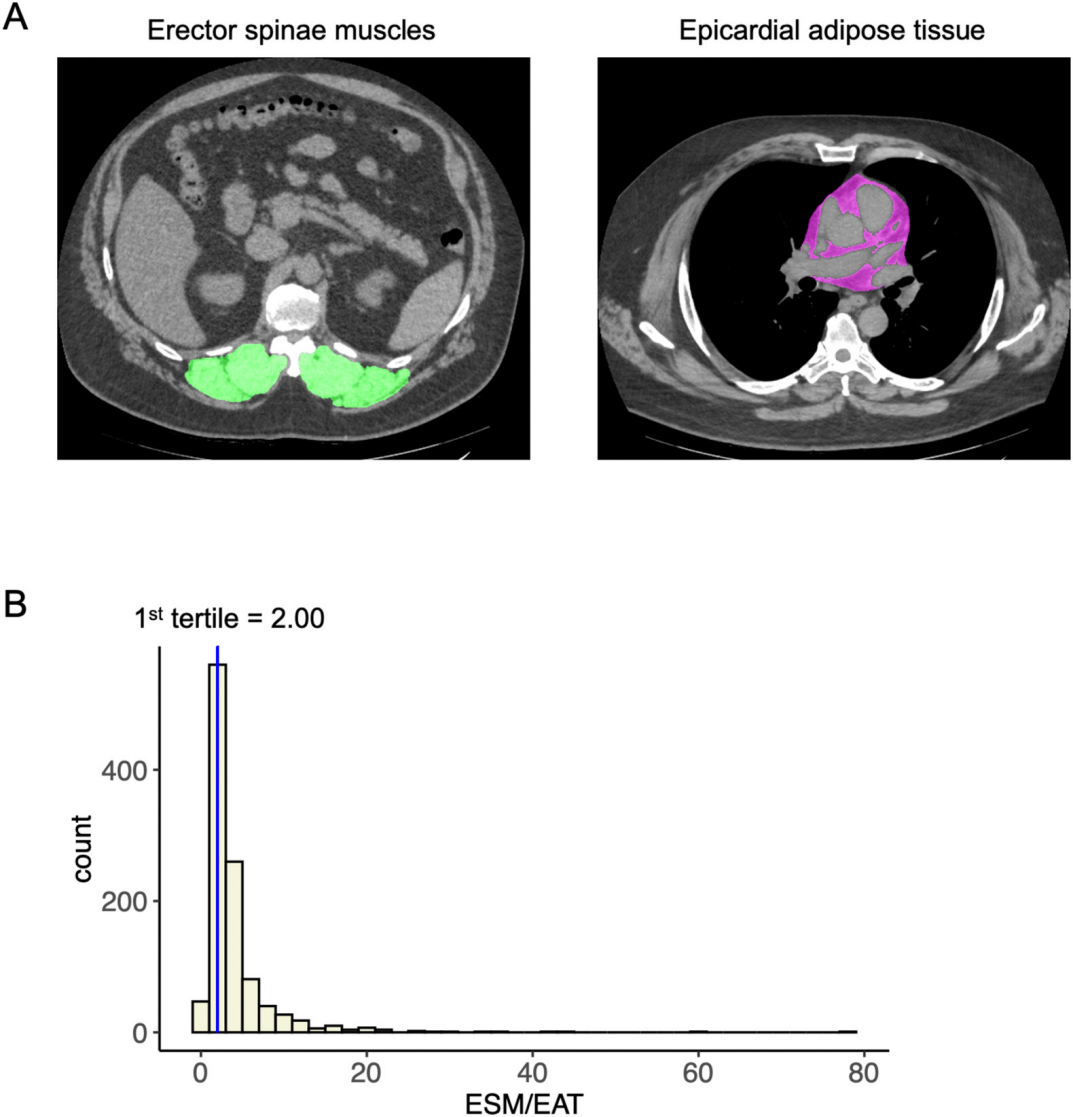
Representative CT images used to measure each muscle and adipose tissue. (A) Representative CT images used to measure erector spinae muscle (ESM) (green) and epicardial adipose tissue (EAT) (pink). (B) Distribution of ESM/EAT ratio and definition of high and low ESM/EAT group (first tertile of values).

ESM was quantitatively evaluated using ImageJ (Fiji) software for manual masking of regions of interest and custom‐made Python scripts for automatic calculation of areas, as previously reported [3, 14]. The left and right ESM were manually segmented from areas with CT values ranging from −50 HU to + 90 HU on a single axial slice of the inferior margin of the 12th thoracic vertebra [15]. The mean attenuation of the ESM, an indicator of skeletal muscle quality, was quantified as the CT density of the ESM [16]. The EAT region at the origin of the left main coronary artery, closely related to the total volume of the entire EAT [2, 17], was located by manually tracing the pericardium and extracting regions with CT values between −230 and −30 HU (Figure 2A).

The psoas muscles were manually segmented on the central axial slice of the third lumbar vertebra, with CT values ranging from −50 HU to + 90 HU [7]. Abdominal tissue was collected from the central slice of the third lumbar vertebra, with total abdominal adipose tissue or abdominal visceral tissue (Abd‐VAT) measured using commercially available software (SYNAPSE VINCENT software, FUJIFILM, Tokyo, Japan) [7, 18]. The same level of abdominal subcutaneous adipose tissue (Abd‐SAT) was obtained by subtracting Abd‐VAT from the total abdominal tissue (Figure S1A). Abdominal CT was performed simultaneously with chest CT within the first 48 h of admission. Segmentation of pneumonia was accomplished using commercial software (SYNAPSE VINCENT software, FUJIFILM, Tokyo, Japan) [11]. If coronary artery calcium (CAC) was detected, the Agatston CAC scores were calculated quantitatively using ImageJ (Fiji) software for manual masking of the region of interest and custom‐made Python scripts for automatic calculation of the region [19].

### 2.5 Evaluation of Muscle‐to‐Fat Ratio

The ESM/EAT ratio served as an indicator of muscle atrophy. In oncology research, the muscle‐to‐fat ratio is often assessed using the combination of CT‐derived psoas muscle and visceral abdominal fat [7]. Therefore, we confirmed the correlation between abdominal CT measurements of the psoas muscle/visceral fat ratio and chest CT measurements of the ESM/EAT ratio (*ρ* = 0.79, *p* < 0.001) (Figure S1B).

### 2.6 Statistical Analysis

For the baseline variables, categorical variables were presented as frequencies and proportions, and continuous variables as means and standard deviations. Data were compared using chi‐square tests for categorical variables and *t*‐tests for continuous variables. Spearman's rank correlation coefficient was calculated to examine the relationship between the ESM/EAT and psoas/visceral fat ratios or laboratory findings. The area under receiver operating characteristic (ROC) curve (AUC) was used to predict critical outcomes, and the cutoff value was determined using the Youden index. The cutoff value was then used in multivariable logistic regression analyses to investigate associations with critical outcomes. The models were adjusted for patient characteristics, such as age, sex, BMI, smoking history, comorbidities (hypertension, diabetes mellitus, cardiovascular disease and chronic kidney disease) and pneumonia volume [13, 20]. Adjusted odds ratios (aOR) are shown with 95% confidence intervals (CI). Statistical significance was set at *p* < 0.05. All statistical analyses were performed using the JMP 17 Pro software (SAS Institute Japan Ltd., Tokyo, Japan).

## 3 Results

### 3.1 Patient Characteristics

The distribution of ESM/EAT ratios is shown in Figure 2B, with a normal distribution and a median of 2.69. Although both ESM and EAT were higher in male, no significant difference was observed in the ESM/EAT ratio between male and female (4.35 vs. 3.92, *p* = 0.20). According to the first tertile of 2.00, patients were classified into the high ESM/EAT group (*n* = 721) and low ESM/EAT group (*n* = 353).

### 3.2 Comparison of Clinical Characteristics of the Two Groups Stratified by ESM/EAT Ratio

A comparison of the clinical characteristics between the high and low ESM/EAT groups is shown in Table 1. Patients in the low ESM/EAT group were older, had a higher BMI, were more frequent smokers and had a higher prevalence of hypertension, diabetes mellitus, cardiovascular disease, hyperuricaemia and chronic kidney disease than those in the high ESM/EAT group.

**TABLE 1:** Comparison of the backgrounds of patients with COVID‐19 between high/low ESM/EAT groups.

Parameter	Total	High ESM/EAT	Low ESM/EAT	*p*
	(*n* = 1074)	(*n* = 721)	(*n* = 353)	
Age, mean (SD)	55.4 (16.5)	51.4 (16.3)	63.4 (13.6)	< 0.001
Sex, Male, *n* (%)	741 (68.9)	504 (69.9)	237 (67.1)	0.36
BMI, mean (SD)	24.3 (6.2)	24.3 (6.6)	26.3 (4.8)	< 0.001
Ex/Current smoking, *n* (%)	488 (46.0)	312 (43.8)	176 (50.4)	0.043
**Comorbidities**				
Hypertension, *n* (%)	334 (31.2)	164 (22.8)	170 (48.3)	< 0.001
Diabetes mellitus, *n* (%)	204 (19.1)	104 (14.5)	100 (28.3)	< 0.001
Cardiovascular disease, *n* (%)	103 (9.6)	45 (6.3)	59 (16.6)	< 0.001
Autoimmune disease, *n* (%)	70 (6.6)	42 (5.9)	28 (8.0)	0.19
Malignancy, *n* (%)	100 (9.3)	63 (8.8)	37 (10.5)	0.37
COPD, *n* (%)	29 (2.7)	17 (2.4)	12 (3.5)	0.32
Asthma, *n* (%)	81 (7.7)	50 (7.1)	31 (8.9)	0.33
Hyperuricaemia, *n* (%)	116 (11.1)	60 (8.4)	56 (16.1)	< 0.001
Chronic liver disease, *n* (%)	31 (2.9)	18 (2.5)	13 (3.7)	0.33
Chronic kidney disease, *n* (%)	86 (8.1)	42 (5.9)	44 (12.6)	< 0.001

*Note:* Data are presented as *n* (%) or mean (standard deviation).

Abbreviations: BMI, body mass index; COPD, chronic obstructive pulmonary disease.

A comparison of laboratory findings between the two groups is shown in Table 2. Patients in the low ESM/EAT group had higher white blood cell, neutrophil counts, C‐reactive protein (CRP), ferritin, lactate dehydrogenase (LDH), creatinine and Krebs von den Lungen‐6 (KL‐6) levels than those in the High ESM/EAT group.

**TABLE 2:** Comparison of laboratory findings in the two groups.

Parameter	Total	High ESM/EAT	Low ESM/EAT	*p*
	(*n* = 1074)	(*n* = 721)	(*n* = 353)	
White blood cells, ×10^3^/μL	5.35 (2.35)	5.17 (2.29)	5.71 (2.41)	< 0.001
Neutrophils, ×10^3^/μL	3.80 (2.09)	3.60 (1.94)	4.21 (2.31)	< 0.001
Lymphocytes, ×10^3^/μL	1.07 (0.49)	1.08 (0.49)	1.04 (0.50)	0.22
Eosinophils, /μL	21.1 (57.1)	19.3 (47.8)	24.6 (72.5)	0.16
Haemoglobin, g/dL	14.2 (1.91)	14.3 (1.86)	14.0 (2.02)	0.004
Platelets, ×10^4^/μL	19.1 (7.09)	19.1 (6.78)	19.4 (7.69)	0.41
Albumin, g/dL	3.73 (0.53)	3.80 (0.53)	3.59 (0.51)	< 0.001
T‐Bil, mg/dL	0.67 (0.40)	0.66 (0.44)	0.69 (0.32)	0.2
AST, IU/L	39.2 (31.8)	36.6 (31.1)	44.6 (32.4)	< 0.001
ALT, IU/L	37.0 (34.1)	34.6 (32.3)	42.1 (37.0)	< 0.001
ALP, IU/L	111 (88)	106 (76)	120 (106)	0.014
γ‐GTP, IU/L	65.5 (75.7)	58.0 (66.2)	80.9 (90.1)	< 0.001
BUN, mg/dL	16.8 (13.1)	15.7 (12.3)	19.1 (14.5)	< 0.001
Creatinine, mg/dL	1.26 (2.13)	1.14 (1.87)	1.52 (2.56)	0.005
LDH, IU/L	262 (109)	250 (100)	288 (122)	< 0.001
UA, mg/dL	4.85 (1.65)	4.70 (1.54)	5.15 (1.83)	< 0.001
CK, IU/L	177 (571)	183 (666)	166 (293)	0.65
BNP, pg/mL	42.6 (216.3)	31.6 (214.8)	63.8 (218.1)	0.06
Ferritin, ng/mL	577 (786)	531 (753)	674 (844)	0.007
HbA1c, %	6.25 (1.20)	6.11 (1.16)	6.55 (1.25)	< 0.001
Fibrinogen, mg/dL	474 (141)	462 (136)	499 (148)	< 0.001
D‐dimer, μg/mL	1.64 (4.20)	1.50 (3.91)	1.92 (4.73)	0.12
CRP, mg/dL	4.69 (6.03)	3.92 (5.51)	6.26 (6.71)	< 0.001
PCT, ng/mL	0.28 (1.32)	0.28 (1.46)	0.29 (0.98)	0.88
KL‐6, U/mL	291 (267)	265 (175)	345 (390)	< 0.001

*Note:* Data are presented as mean (standard deviation).

Abbreviations: γ‐GTP, gamma‐glutamyl transferase; ALT, alanine aminotransferase; ALP, alkaline phosphatase; AST, aspartate aminotransferase; BMI, body mass index; BNP, brain natriuretic peptide; BUN, blood urea nitrogen; CK, creatine kinase; CRP, C‐reactive protein; HbA1c, haemoglobin A1c; KL‐6, Krebs von den Lungen‐6; LDH, lactate dehydrogenase; PCT, procalcitonin; T‐Bil, total bilirubin; UA, uric acid.

The ESM/EAT ratio showed a weak negative correlation with leukocyte and neutrophil counts, neutrophil‐to‐lymphocyte ratio (NLR), LDH, ferritin and CRP. Moreover, the ESM/EAT ratio demonstrated a positive correlation with ESM density (Table 3).

**TABLE 3:** Correlation of ESM/EAT ratio with laboratory parameters and CT indices.

Parameter	*ρ*	*p*
White blood cells	−0.16	< 0.001
Neutrophils	−0.19	< 0.001
NLR	−0.18	< 0.001
LDH	−0.26	< 0.001
Ferritin	−0.18	< 0.001
CRP	−0.28	< 0.001
ESM density	0.45	< 0.001
Agatston score	−0.18	< 0.001

**Abbreviations**: CRP, C‐reactive protein; LDH, lactate dehydrogenase; NLR, neutrophil‐to‐lymphocyte ratio.

### 3.3 Comparison of Clinical Outcomes Between Two Groups

**FIGURE 3:**
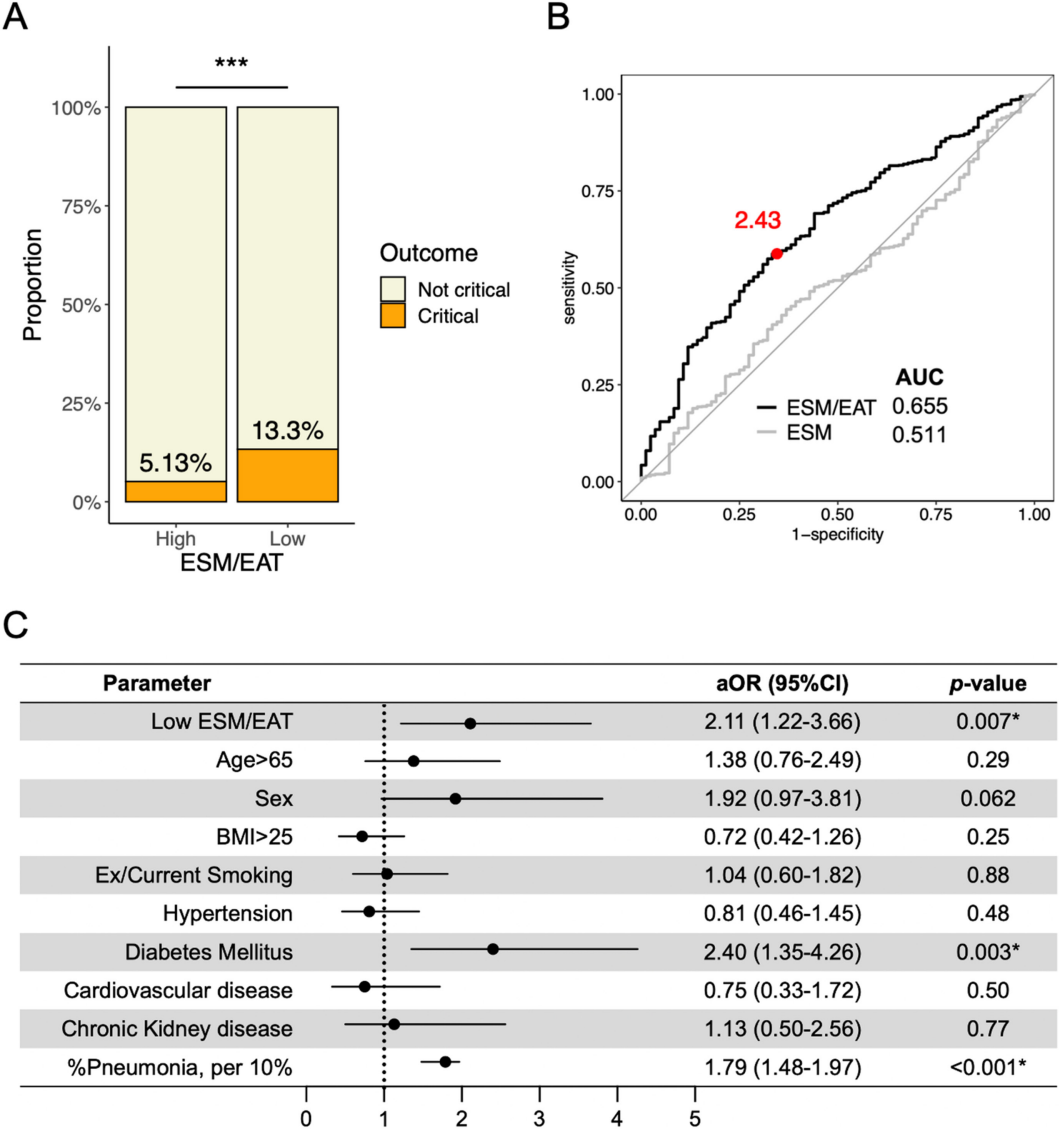
Relationship between ESM/EAT ratio and disease severity in hospitalized patients with COVID‐19. (A) Comparison of the prevalence of critical outcomes by high and low ESM/EAT. (B) Receiver operating characteristic curve analysis for critical outcome based on ESM/EAT ratio and ESM. (C) Multivariable logistic regression analysis for the association between the critical outcomes and low ESM/EAT ratio and already‐known risk factors of COVID‐19. aOR, adjusted odds ratio; AUC, area under the curve; BMI, body mass index; CI, confidence interval. *, Significant; ***, *p* < 0.001.

A comparison of the clinical characteristics between the high and low ESM/EAT groups is shown in Figures 3A and S2. Patients in the low ESM/EAT group had higher rates of critical outcomes (13.3% vs. 5.13%, *p* < 0.001), mortality (2.55% vs. 0.69%, *p* = 0.019) and use of invasive positive pressure ventilation (IPPV) (4.25% vs. 1.11%, *p* = 0.001) and high‐flow nasal cannula (HFNC) (7.93% vs. 3.74%, *p* = 0.005) than those in the high ESM/EAT group. Furthermore, the proportion of critical outcomes was consistently higher in the low ESM/EAT group than in the high ESM/EAT group in both male (15.2% vs. 6.35%, *p* < 0.001) and female (9.48% vs. 2.30%, *p* = 0.006) (Figure S3). In addition, stratification by epidemic wave revealed that patients in the low ESM/EAT group had a higher incidence of critical outcomes from the first to the third epidemic wave (Figure S4). Moreover, the ESM/EAT ratio showed a weak negative correlation with the length of hospital stay, with patients in the low ESM/EAT group experiencing a longer hospital stay compared to those in the high ESM/EAT group (Figure S5). On the other hand, there was no significant difference in posthospitalization complications between the two groups (Figure S6).

**FIGURE 4:**
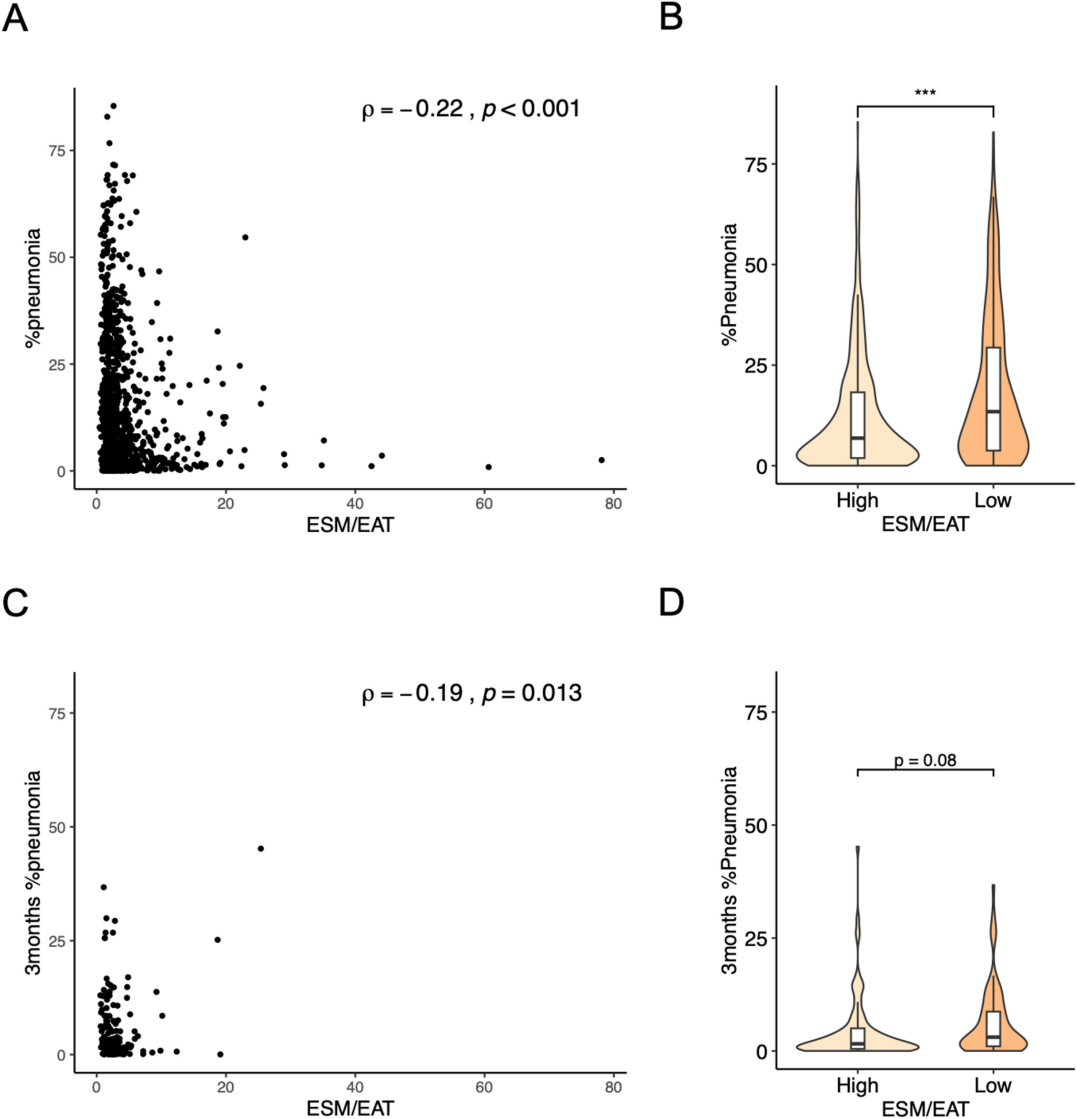
Association between ESM/EAT ratio and pneumonia volume. (A) Correlation between ESM/EAT ratios and pneumonia volume. (B) Comparison of pneumonia volume by high and low ESM/EAT. ***, *p* < 0.001. (C) Correlation between ESM/EAT ratios and residual pneumonia volume at 3 months after onset. (D) Comparison of residual volume at 3 months after onset by high and low ESM/EAT.

Furthermore, we examined the relationship between the ESM/EAT ratio and pneumonia volume using AI‐based quantification, as shown in Figure 4 and Table 4. The ESM/EAT ratio exhibited a weak negative correlation with pneumonia volume; patients with lower ESM/EAT ratios had a greater pneumonia volume at admission. Multivariable analysis confirmed a significant association between the ESM/EAT ratio and pneumonia volume. In addition, the ESM/EAT ratio was weakly negatively correlated with residual pneumonia volume at 3 months after onset. Patients in the low ESM/EAT group tended to have larger residual pneumonia volumes at this follow‐up point.

**TABLE 4:** Multivariable analysis for the association between pneumonia volume and low ESM/EAT ratio and already‐known risk factors of COVID‐19.

Parameter	*β*	*p*
Low ESM/EAT	0.12	< 0.001
Age > 65	−0.007	0.85
Sex	0.05	0.12
BMI > 25	0.095	0.005
Ex/Current smoking	−0.032	0.31
Hypertension	−0.009	0.8
Diabetes mellitus	0.031	0.35
Cardiovascular disease	0.007	0.83
Chronic kidney disease	0.044	0.18

Abbreviation: BMI, body mass index.

Subsequently, the ROC curve analysis determined the optimal cutoff point for predicting critical outcomes to be 2.43, with a sensitivity of 66.7%, specificity of 58.8% and an AUC of 0.655. This AUC was higher than that of ESM alone, indicating that the ESM/EAT ratio provides better predictive accuracy (Figure 3B). Using an ESM/EAT cutoff of 2.43, a low ESM/EAT ratio was independently associated not only with pneumonia volume (Table 4) but also with critical outcomes, even after adjusting for pneumonia volume and other known severity factors (aOR: 2.11, 95% CI: 1.22–3.66) (Figure 3C). However, this association did not reach statistical significance for the length of hospital stay (Table S1). The ROC curve analysis demonstrated that the ESM/EAT ratio was particularly effective in predicting mortality and the use of IPPV, with the highest AUC for IPPV prediction at 0.737 (Figure S2D).

## 4 Discussion

To the best of our knowledge, this study is the first to report on the effect of reduced muscle mass and visceral fat accumulation on COVID‐19. We found that muscle loss and increased fat storage, assessed using chest CT, were significantly associated with critical outcomes. The strength of this study lies in its long study period and large patient cohort, showing that the ESM/EAT ratio is associated with critical outcomes, even in multivariable analysis using preexisting severity factors and quantitative pneumonia volume. Given the widespread use of chest CT in COVID‐19 clinical settings, the extent of pneumonia is often reported to be related to disease severity [11]. The prognostication of muscle loss accompanied by fat accumulation independent of pneumonia volume, as demonstrated by our findings, underscores the significance of a detailed assessment of body composition in COVID‐19 practice and may contribute to clinicians' risk management.

In this study, the low ESM/EAT group exhibited worse outcomes than the high ESM/EAT group across various outcomes, including critical outcomes and mortality. Several factors could explain this association. First, patients with low ESM/EAT might have characteristics amplifying the severity of COVID‐19, including lifestyle‐related diseases and advanced age [20, 21]. The results of this study are consistent with previous studies, indicating an association between muscle loss with fat accumulation and lifestyle‐related diseases such as diabetes mellitus and hypertension in the general population [22]. However, the robust relationship between ESM/EAT and critical outcomes observed in multivariable analysis encompassing these variables as covariates suggests that ESM/EAT has additional prognostic impact over these clinical characteristics. Second is the association between the ESM/EAT ratio and pneumonia volume. Patients with a low ESM/EAT ratio presented with significantly larger pneumonia volumes at admission, and this association remained significant even after adjusting for existing severity factors in a multivariable analysis. Given that pneumonia severity is strongly linked to poor outcomes [11], this suggests that the ESM/EAT ratio may be particularly relevant for predicting respiratory outcomes. Importantly, even after accounting for pneumonia volume in the multivariable analysis, the ESM/EAT ratio continued to be significantly associated with clinical outcomes, highlighting its added value as a predictive marker alongside quantitative pneumonia assessments. Third, ESM and EAT serve as composite indices, integrating factors potentially linked to COVID‐19 severity. Considering the ESM perspective, ESM reflects not only an antigravity muscle, the level of physical activity [23]. In fact, previous reports have shown that ESM can predict prognosis and low physical activity levels in patients with COPD [24]. Therefore, low ESM may reflect a low level of physical activity and consequently contribute to a poor prognosis. Additionally, considering the EAT perspective, its adjacency to the pulmonary artery suggests a potential conduit for the direct diffusion of inflammatory mediators into the pulmonary circulation [25]. Hence, EAT may exert a direct local effect on adjacent lungs through paracrine or vascular‐mediated secretory mechanisms, resulting in the potentiation of a systemic inflammatory response [2, 26, 27]. As these metrics represent the ratios of ESM to EAT, they serve as acute indicators of COVID‐19. Fourth, the ESM/EAT ratio exhibited a positive correlation with ESM density. Our prior research indicates that lower ESM density is linked to increased severity of COVID‐19 [3]. This suggests that a low ESM/EAT ratio may be associated with deteriorated muscle health and greater disease severity.

Previous studies have demonstrated that reduced muscle mass and visceral fat accumulation are linked to the severity of COVID‐19 [28, 29]. In our analysis, the ratio between muscle and fat—indicating a relative decrease in muscle mass compared to fat—was more strongly associated with COVID‐19 outcomes than muscle mass reduction alone. This result aligns with existing studies. Muscle loss with fat accumulation has been reported to elicit systemic inflammation and insulin resistance [30], consistent with our findings that the ESM/EAT ratio correlates with systemic inflammatory markers. In addition, a previous report showed that the quantitative assessment of pneumonia in COVID‐19 was correlated with the level of systemic inflammation [11], thereby rationalizing the modest correlation between the ESM/EAT ratio and pneumonia extent in our findings. Moreover, the ROC curves revealed that the ESM/EAT ratio is associated especially with mortality and IPPV usage. Because the ESM/EAT ratio was associated with pneumonia volume and not with after hospitalization complications, the ESM/EAT ratio may serve as a biomarker for predicting COVID‐19 pneumonia. Nevertheless, the association between ESM/EAT and critical outcomes remains independent of pneumonia extent, underscoring the necessity of incorporating ESM/EAT evaluation along with pneumonia volume assessment to identify patients with severe illness. Notably, previous reports suggest that severe COVID‐19 manifestations, including severe pneumonia, heightened inflammation and cytokine storms can occur in the later posthospitalization period, approximately 10 days after the onset of illness [31]. It is plausible that ESM/EAT may exacerbate pneumonia post‐hospitalization, given the absence of an association between ESM/EAT and posthospital complications in our study, contrasting with its correlation with pneumonia volume and inflammatory response.

In this study, the association between the ESM/EAT ratio and clinical outcomes was most pronounced during the first through third waves of the pandemic. Although the severity of COVID‐19 has decreased over time because of viral mutations, advancements in treatment and widespread vaccination, more severe cases were prevalent during the earlier waves, which may have enhanced the sensitivity in detecting the utility of biomarkers [32]. Our analysis of residual pneumonia area at 3 months after onset found that patients with a low ESM/EAT ratio were more likely to have larger residual pneumonia volumes. Given that persistent lung shadows are significant in the context of long‐term sequelae [33], the ESM/EAT ratio may serve as a biomarker for predicting both acute‐phase outcomes and long‐term pulmonary consequences.

This study used ESM/EAT rather than ESM or EAT. In daily clinical practice, particularly in situations with a high number of patients, such as during the COVID‐19 pandemic, it is challenging to have the same CT scanner, analysis software and analysts to consistently conduct image analysis. This can lead to variability in single‐organ area measurements. However, the use of two‐organ ratios from the same image may reduce this variability, making it more broadly acceptable as a clinical indicator. Although reference values for muscle mass have been proposed for abdominal CT [34], they have not been established for chest CT. Therefore, it would be useful to determine the ratio to fat in the same patient. Moreover, low ESM and high EAT have been associated with poor outcomes across various diseases [35, 36], indicating that the ESM/EAT ratio may have broader applications beyond COVID‐19. Our results indicate an optimal ESM/EAT cutoff of 2.43 for predicting critical outcomes. However, because of ethnic differences in body composition and the clinical course of COVID‐19 [37], validation in other ethnic groups is desirable.

This study had several limitations. First, the use of various CT models may have affected the results. Second, the inclusion of only hospitalized patients may have introduced a selection bias. Moreover, patients who were hospitalized but did not undergo CT scans were excluded, which may have limited the evaluation, particularly those who were mildly ill at admission. Third, this study did not assess the effects of vaccination or viral variants on outcomes. In the analysis by pandemic wave, the correlation between the ESM/EAT ratio and COVID‐19 severity weakened after the fourth wave. The evolving clinical characteristics of COVID‐19 patients, influenced by vaccination and virus variants, likely introduced bias and impacted the results. Fourth, the use of fully automated AI solutions for CT analysis is becoming increasingly mainstream in quantification studies [38]. However, the body composition evaluation in this study was semiautomated, presenting limitations for its practical implementation. Fifth, this study did not evaluate muscle strength or daily activity, which are key components in defining sarcopenic obesity [39]. Although patients with a low ESM/EAT ratio may include those with sarcopenic obesity, it was challenging to explore this in detail within the study. Future research should focus on the impact of sarcopenic obesity on COVID‐19, including assessments of muscle strength and daily activity.

In conclusion, the low ESM/EAT ratio in COVID‐19 patients can predict critical outcomes. Furthermore, the low ESM/EAT ratio was associated with poor outcomes independent of pneumonia severity. These findings underscore the importance of appropriate body composition assessments and risk stratification in COVID‐19 patients.

## Author Contributions

**Takashi Shimada:** conceptualization, data curation, formal analysis, methodology, resource, visualization, writing – original draft. **Tomoki Maetani:** conceptualization, formal analysis, methodology, software, visualization, writing – review and editing. **Shotaro Chubachi:** conceptualization, data curation, formal analysis, methodology, project administration, resource, software, supervision, visualization, writing – review and editing. **Naoya Tanabe:** conceptualization, formal analysis, methodology, project administration, software, supervision, visualization, writing – review and editing. **Takanori Asakura:** formal analysis, methodology, supervision, visualization, writing – review and editing. **Ho Namkoong:** data curation, methodology, supervision, writing – review and editing. **Hiromu Tanaka:** data curation, methodology, resource. **Shuhei Azekawa:** data curation, resource. **Shiro Otake:** data curation, resource. **Kensuke Nakagawara:** data curation, resource. **Takahiro Fukushima:** data curation, resource. **Mayuko Watase:** data curation, resource. **Yusuke Shiraishi:** conceptualization, formal analysis, methodology, software, visualization, writing – review and editing. **Hideki Terai:** formal analysis, supervision, writing – review and editing. **Mamoru Sasaki:** formal analysis, resource, writing – review and editing. **Soichiro Ueda:** formal analysis, resource, writing – review and editing. **Yukari Kato:** formal analysis, resource, writing – review and editing. **Norihiro Harada:** formal analysis, resource, writing – review and editing. **Shoji Suzuki:** formal analysis, resource, writing – review and editing. **Shuichi Yoshida:** formal analysis, resource, writing – review and editing. **Hiroki Tateno:** formal analysis, resource, writing – review and editing. **Kaoruko Shimizu:** formal analysis, resource, writing – review and editing. **Susumu Sato:** formal analysis, resource, writing – review and editing. **Yoshitake Yamada:** supervision, writing – review and editing. **Masahiro Jinzaki:** supervision, writing – review and editing. **Toyohiro Hirai:** supervision, writing – review and editing. **Yukinori Okada:** supervision, writing – review and editing. **Ryuji Koike:** supervision, writing – review and editing. **Makoto Ishii:** supervision, writing – review and editing. **Akinori Kimura:** supervision, writing – review and editing. **Seiya Imoto:** supervision, writing – review and editing. **Satoru Miyano:** supervision, writing – review and editing. **Seishi Ogawa:** supervision, writing – review and editing. **Takanori Kanai:** supervision, writing – review and editing. **Koichi Fukunaga:** supervision, writing – review and editing.

## Ethics Statement

This study was approved by the ethics committee of the Keio University School of Medicine (20200061) and related research institutions. All the participants provided informed consent.

## Conflicts of Interest

The authors declare no conflicts of interest.

## Supporting information


**Figure S1** Representative CT images of the psoas muscle and abdominal visceral adipose tissue, and the correlation between abdominal and chest imaging indices. (a) Representative CT images used to measure the psoas muscle (orange), abdominal subcutaneous adipose tissue (blue), and abdominal visceral adipose tissue (Abd‐VAT; red). (b) Correlation between ESM/EAT ratios and the psoas muscle/Abd‐VAT.

**Figure S2** Comparison of the prevalence of (a) mortality, (b) invasive positive pressure ventilation (IPPV), and (c) high‐flow nasal cannula (HFNC) by high and low ESM/EAT. (d) Comparison of ROC curves for individual outcomes (mortality, IPPV, and HFNC). *, *p* < 0.05; **, *p* < 0.01.

**Figure S3** Comparison of the prevalence of critical outcomes by high and low ESM/EAT stratified by sex. **, p < 0.01; ***, *p* < 0.001.

**Figure S4** Comparison of critical outcomes by high and low ESM/EAT ratio, stratified by epidemic wave ((a) first to third, (b) fourth, (c) fifth, and (d) sixth to seventh waves). **, p < 0.01; NS., Not significant.

**Figure S5** Association between ESM/EAT ratio and length of hospital stay. (a) Correlation between ESM/EAT ratios and the length of hospital stay. (b) Comparison of the length of hospital stay by high and low ESM/EAT. ***, p < 0.001.

**Figure S6** Comparison of the incidence of posthospitalization complications between the high and low ESM/EAT groups. NS., Not significant.

**Table S1** Multivariable analysis for the association between the length of hospital stay and low ESM/EAT ratio and already‐known risk factors of COVID‐19.
