# Supplementary figures and images for: Erector Spinae Muscle to Epicardial Visceral Fat Ratio on Chest CT Predicts the Severity of Coronavirus Disease 2019
Source: J Cachexia Sarcopenia Muscle. 2025 Jan 27;16(1):e13721. doi: 10.1002/jcsm.13721 (PMC11770476; doi:10.1002/jcsm.13721)

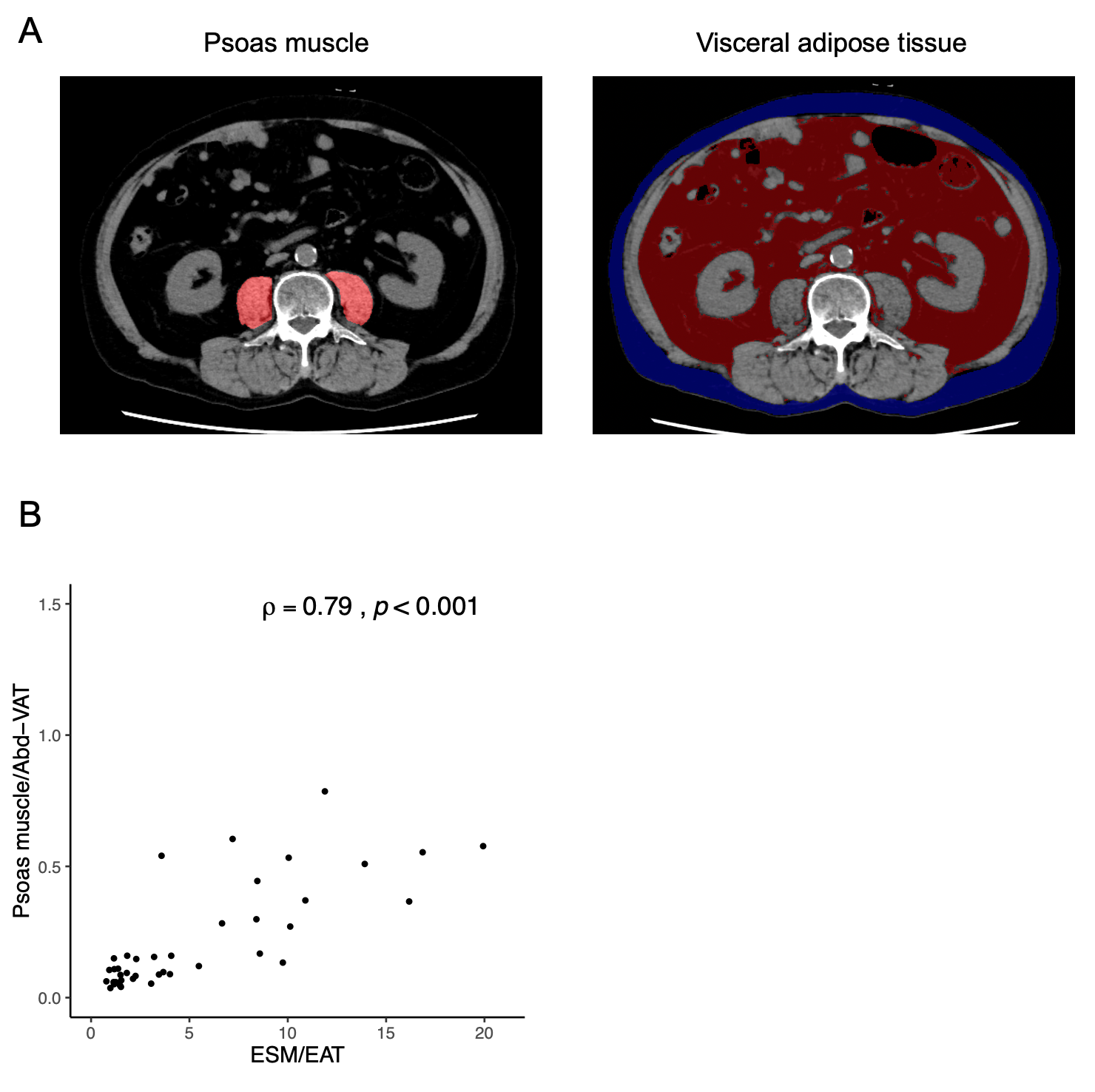

Supplement: Supplementary file 1 — Figure S1 Representative CT images of the psoas muscle and abdominal visceral adipose tissue, and the correlation between abdominal and chest imaging indices. (a) Representative CT images used to measure the psoas muscle (orange), abdominal subcutaneous adipose tissue (blue), and abdominal visceral adipose tissue (Abd‐VAT; red). (b) Correlation between ESM/EAT ratios and the psoas muscle/Abd‐VAT. [file JCSM-16-e13721-s002.tiff]

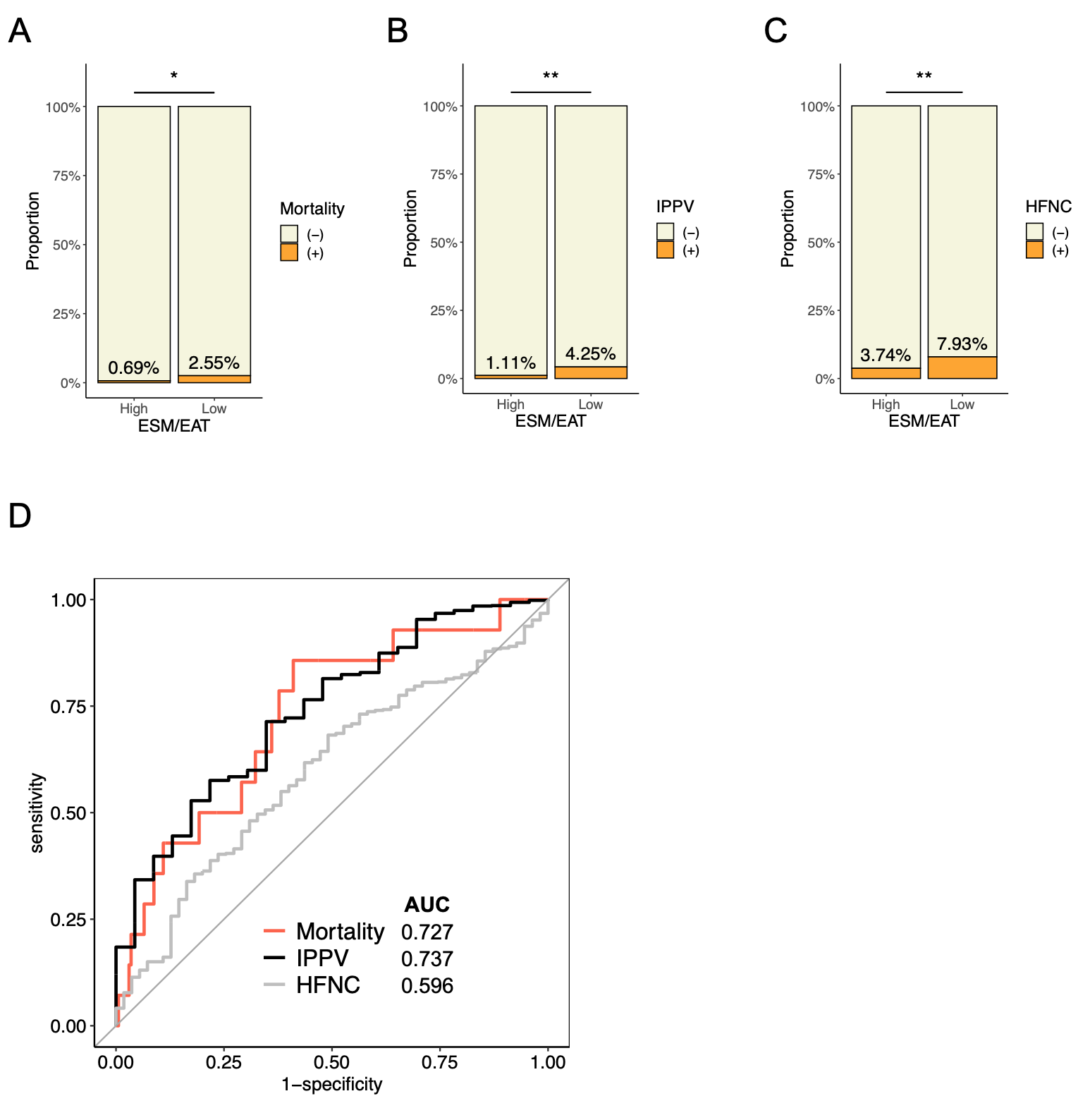

Supplement: Supplementary file 2 — Figure S2 Comparison of the prevalence of (a) mortality, (b) invasive positive pressure ventilation (IPPV), and (c) high‐flow nasal cannula (HFNC) by high and low ESM/EAT. (d) Comparison of ROC curves for individual outcomes (mortality, IPPV, and HFNC). *, p < 0.05; **, p < 0.01. [file JCSM-16-e13721-s007.tiff]

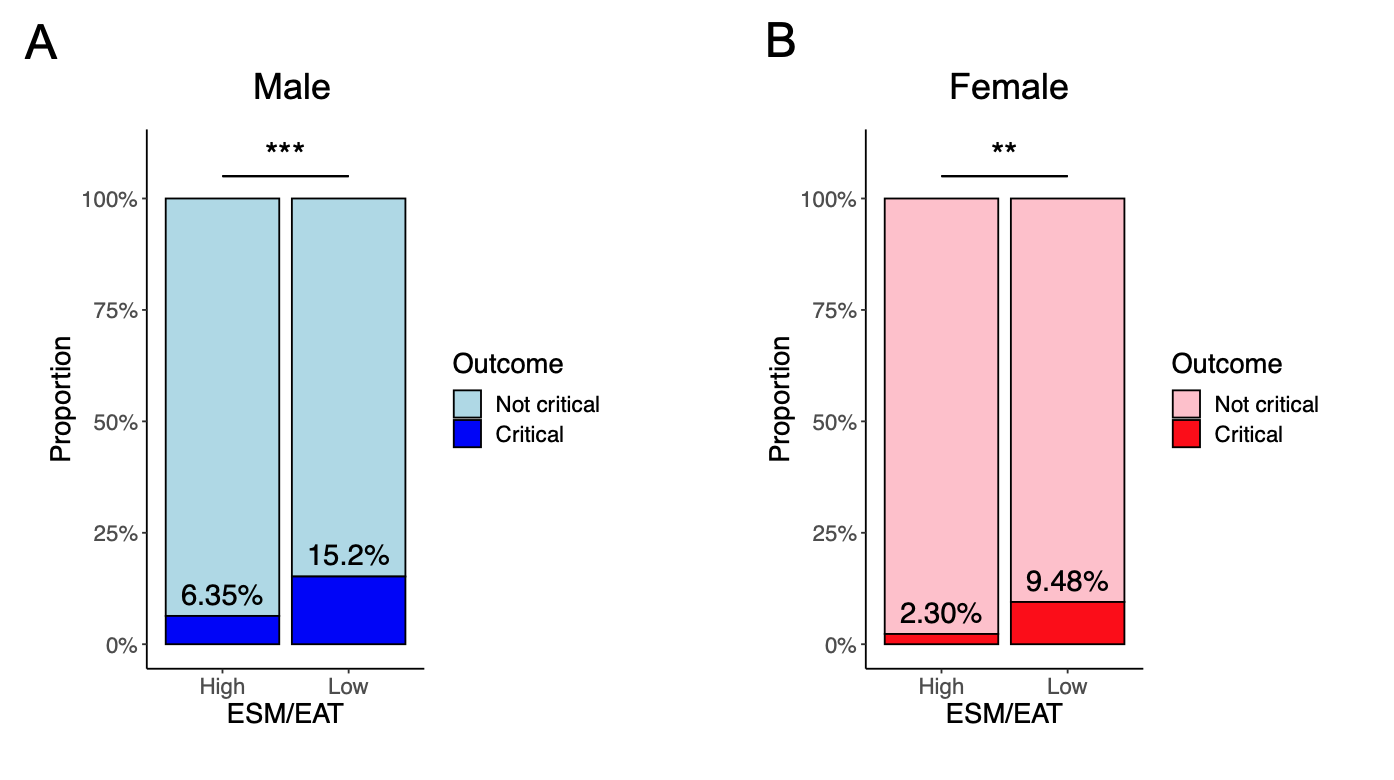

Supplement: Supplementary file 3 — Figure S3 Comparison of the prevalence of critical outcomes by high and low ESM/EAT stratified by sex. **, p < 0.01; ***, p < 0.001. [file JCSM-16-e13721-s005.tiff]

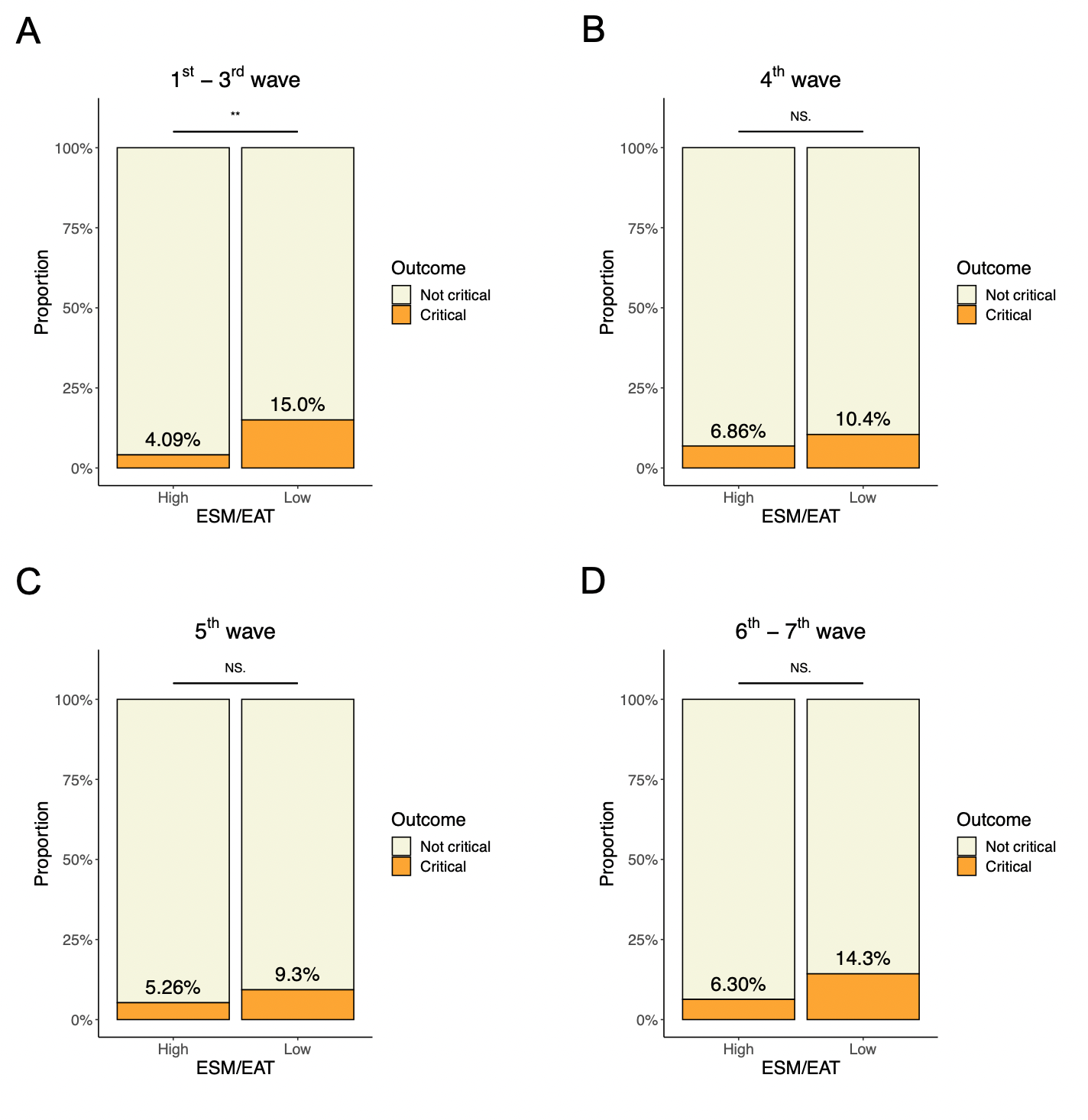

Supplement: Supplementary file 4 — Figure S4 Comparison of critical outcomes by high and low ESM/EAT ratio, stratified by epidemic wave ((a) first to third, (b) fourth, (c) fifth, and (d) sixth to seventh waves). **, p < 0.01; NS., Not significant. [file JCSM-16-e13721-s006.tiff]

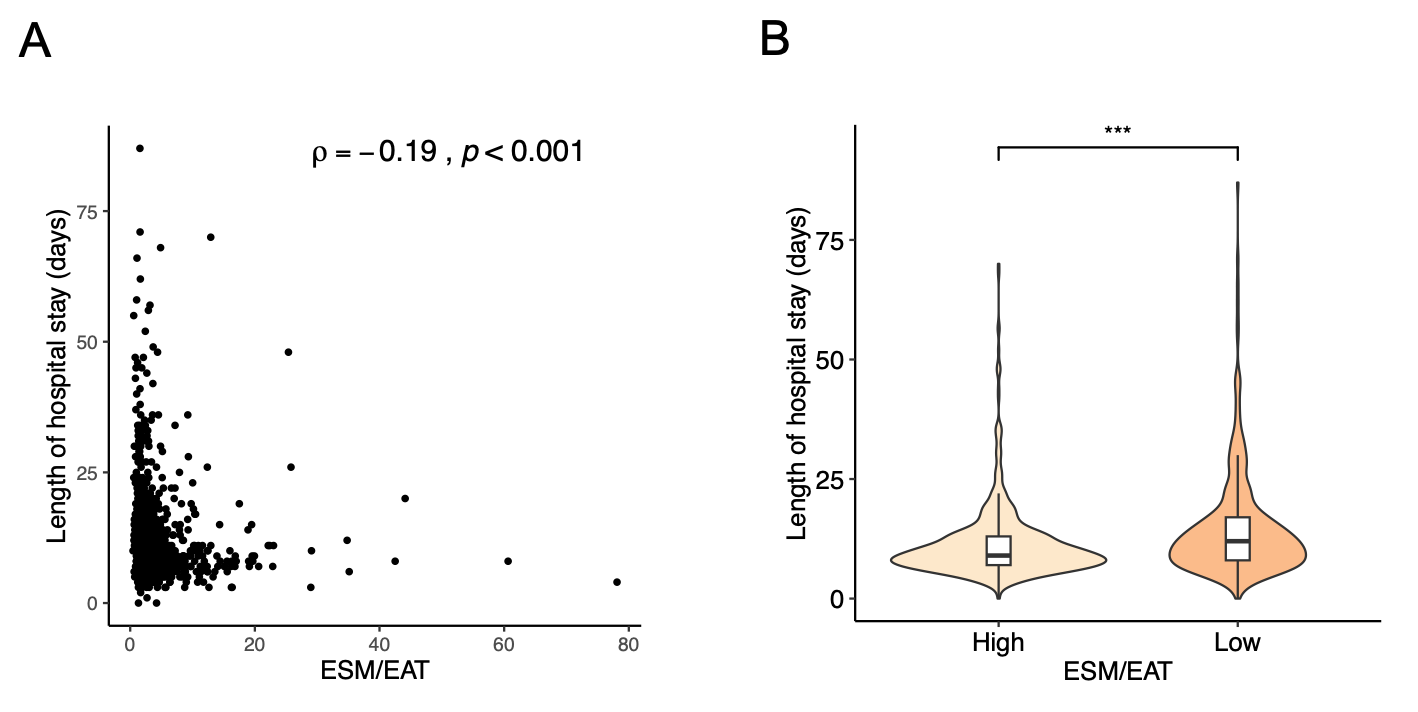

Supplement: Supplementary file 5 — Figure S5 Association between ESM/EAT ratio and length of hospital stay. (a) Correlation between ESM/EAT ratios and the length of hospital stay. (b) Comparison of the length of hospital stay by high and low ESM/EAT. ***, p < 0.001. [file JCSM-16-e13721-s003.tiff]

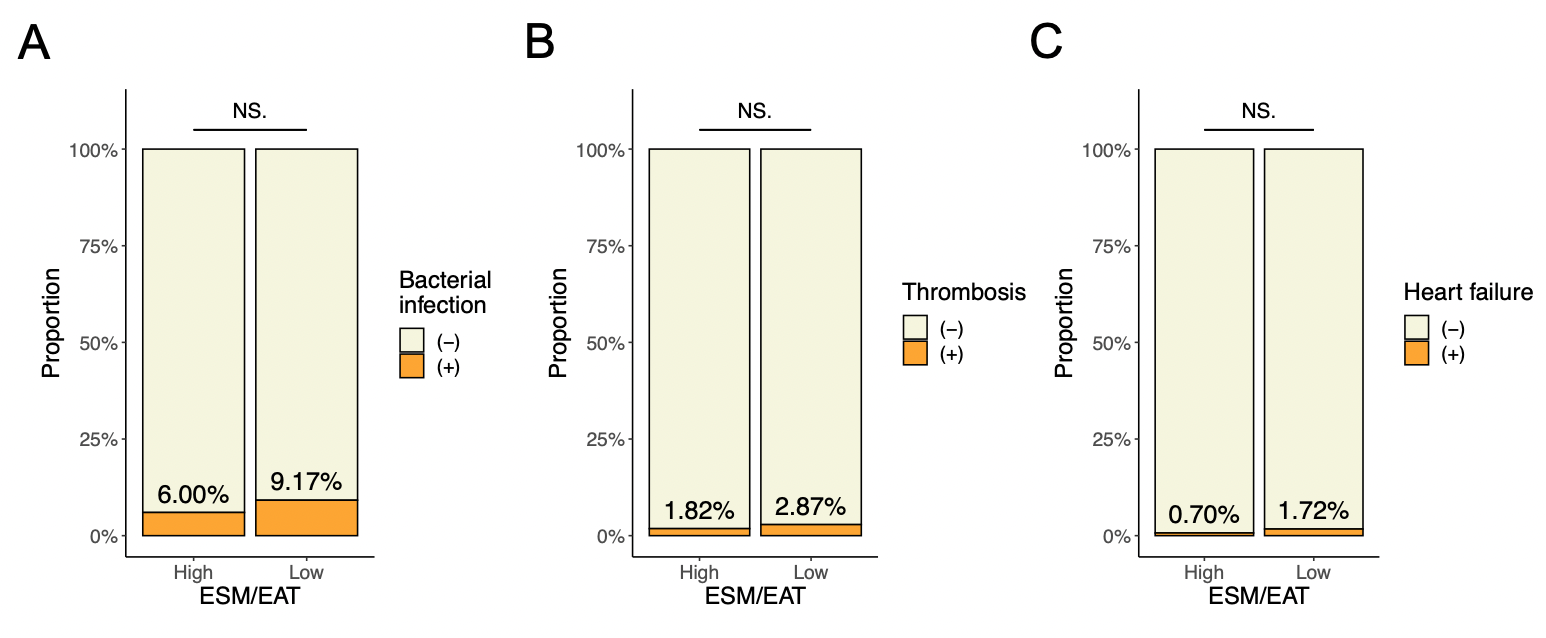

Supplement: Supplementary file 6 — Figure S6 Comparison of the incidence of posthospitalization complications between the high and low ESM/EAT groups. NS., Not significant. [file JCSM-16-e13721-s004.tiff]
